# Growth faltering in rural Gambian children after four decades of interventions: a retrospective cohort study

**DOI:** 10.1016/S2214-109X(16)30355-2

**Published:** 2017-01-17

**Authors:** Helen M Nabwera, Anthony J Fulford, Sophie E Moore, Andrew M Prentice

**Affiliations:** a MRC Unit The Gambia, Banjul, The Gambia; b MRC International Nutrition Group, London School of Hygiene and Tropical Medicine, London, UK; c Division of Women's Health, King's College London, London, UK

## Abstract

**Background:**

Growth faltering remains common in children in sub-Saharan Africa and is associated with substantial morbidity and mortality. Due to a very slow decline in the prevalence of stunting, the total number of children with stunting continues to rise in sub-Saharan Africa. Identification of effective interventions remains a challenge.

**Methods:**

We analysed the effect of 36 years of intensive health interventions on growth in infants and young children from three rural Gambian villages. Routine growth data from birth to age 2 years were available for 3659 children between 1976 and 2012. *Z* scores for weight-for-age, length-for-age, weight-for-length, mid-upper-arm circumference, and head circumference were calculated using the WHO 2006 growth standards. Seasonal patterns of mean *Z* scores were obtained by Fourier regression. We additionally defined growth faltering as fall in *Z* score between 3 months and 21 months of age.

**Findings:**

We noted secular improvements in all postnatal growth parameters (except weight-for-length), accompanied by declines over time in seasonal variability. The proportion of children with underweight or stunting at 2 years of age halved during four decades of the study period, from 38·7% (95% CI 33·5–44·0) for underweight and 57·1% (51·9–62·4) for stunting. However, despite unprecedented levels of intervention, postnatal growth faltering persisted, leading to poor nutritional status at 24 months (length-for-age *Z* score −1·36, 95% CI −1·44 to −1·27, weight-for-age *Z* score −1·20, −1·28 to −1·11, and head circumference *Z* score −0·51, −0·59 to −0·43). The prevalence of stunting and underweight remained unacceptably high (30·0%, 95% CI 27·0–33·0, for stunting and 22·1%, 19·4 to 24·8, for underweight).

**Interpretation:**

A combination of nutrition-sensitive and nutrition-specific interventions has achieved a halving of undernutrition rates, but despite these intensive interventions substantial growth faltering remains. We need to understand the missing contributors to growth faltering to guide development of new interventions.

**Funding:**

UK Medical Research Council, UK Department for International Development.

## Introduction

The combination of fetal growth restriction, underweight, stunting, and wasting in later childhood, suboptimal breastfeeding, and micronutrient deficiencies have been estimated to cause more than 3 million child deaths annually, equivalent to 45% of the global total.^1^ Among these factors, the association between undernutrition and mortality is confounded by the effects of deprivation but is probably at least partly causal as evidenced by the greatly elevated hospital case fatality rates of undernourished children compared with better nourished children.^2^

The Millennium Development Goals (MDGs) adopted underweight as a key indicator for MDG1, but stunting has since been adopted as the preferred indicator because it offers a more stable index of long-term malnutrition. Latest estimates suggest that rates of stunting have been declining in most regions, but there remain 159 million children with stunting worldwide.^3^ The prevalence of stunting has declined most slowly in sub-Saharan Africa, and as a consequence of population growth the absolute number of children with stunting has increased.^3^

Stunting rates fall rapidly as countries pass through the economic transition, but the key elements of progress that alleviate growth faltering are poorly understood, thus limiting the design of interventions and the targeting of health and development inputs in populations that remain impoverished. In this study, we analyse a longitudinal dataset spanning almost four decades of growth monitoring in three rural African villages that have received an unprecedented level of health-orientated interventions. A meta-analysis^4^ of previous interventions for water, sanitation, and hygiene (WASH) has not yielded strong grounds for optimism regarding the likely efficacy of such investments at the levels currently offered. The analysis of randomised trials included more than 4600 children studied over 9–12 months of intervention, and its findings showed no evidence of any beneficial effect on weight-for-age or weight-for-height and only a marginally significant effect on height-for-age of less than a tenth of a standard deviation (0·08 *Z* score, 95% CI 0·00–0·16). Additionally, the *Lancet* Series on Maternal and Child Nutrition^5^ reinforced the conclusion that nutrition interventions alone will have little effect on childhood undernutrition and estimated that, even if scaled up to 90% coverage, the implementation of all of the currently identified evidence-based interventions relating to nutrition would eliminate only about 20% of stunting globally. The results of ongoing trials to test the effect on growth of WASH interventions are keenly awaited.^6^, ^7^

**Research in context**

**Evidence before this study**

We searched PubMed and subsequent reference lists of relevant articles, with combinations of the terms “secular trends in growth”, “growth faltering”, “undernutrition”, “wasting”, “stunting”, “underweight”, “African children”, “rural”, “underfive”, and “infants” between June 1, 2012, and Feb 28, 2015. All studies published between Jan 1, 1980, and Feb 28, 2015, that had the relevant search terms (irrespective of language) were included. The quality of the evidence was inadequate for the research questions that we posed, including what the secular trends were in growth in rural African children younger than 2 years during the past four decades, and how the effect of seasonality on the growth of these children has changed during the past four decades. A small number of longitudinal studies from east and central sub-Saharan Africa described the growth patterns in cohorts of young African children over a period of less than a decade, assessing the effect of seasonality, immunisation uptake, and maternal health factors on the patterns of growth faltering. These findings showed that weight declined after the first 3 months in infants and that improved growth in infancy was associated with immunisation status and indices of adequate maternal nutritional status, whereas the rainy season was associated with reduced growth velocity. However, none of these studies described secular trends in these growth patterns. Additionally, the multicountry analyses used cross-sectional data, making interpretation of trends in growth faltering over time within individual populations difficult. Several studies from southern Africa assessed the secular trends in growth in children of school age and older children (older than 8 years), whereas other researchers combined different cohorts in their analyses, making it difficult to contextualise the associated trends in the social environmental and health interventions within the respective populations.

**Added value of this study**

To our knowledge, this study is the first to describe in fine detail the secular trends in longitudinal and seasonal growth patterns of children in a rural sub-Saharan African community with a constant sampling frame. We have documented the introduction of a series of nutrition-specific and nutrition-sensitive interventions resulting in an unprecedented level of health care in these villages. Simultaneous socioeconomic transitions have occurred with increased access to formal education, employment, and income through remittances from family members overseas. Families have become much less reliant on subsistence farming for their income and nutritional needs. These changes have resulted in reduction of mortality to a tenth of its former level in children younger than 5 years, and major reductions in diarrhoeal and other morbidity. Growth has improved but, despite these profound health and socioeconomic changes, the patterns of childhood growth faltering persist with stunting prevalence remaining at 30%. Our findings indicate that communities must exceed a very high threshold for health and environmental change before growth faltering will be eliminated.

**Implications of all the available evidence**

Children in resource limited settings, particularly in sub-Saharan Africa, continue to have suboptimal growth patterns despite access to public health interventions such as immunisation, clean water, and sanitation. Our analysis suggests that mitigation of growth faltering will need these public health interventions to be combined with many other improvements in children's environments, perhaps including improved housing with the provision of piped water directly into the home. Evidence from countries that have passed through the economic transition suggests that poverty reduction promotes such improvements and is accompanied by rapid declines in stunting.

The implication therefore is that there is a very high threshold for improvements in living conditions, disease elimination, dietary sufficiency, and access to health care that must be exceeded to eliminate malnutrition. On this basis, we predict that current WASH interventions might not be sufficiently intensive to yield a substantial improvement in child growth, and that greater efforts will be required to meet the new UN Sustainable Development Goals (SDGs).^8^ In this study we assessed the aggregate improvements in child growth associated with progressive improvements in a wide range of nutrition-specific and nutrition-sensitive interventions in three rural Gambian villages that have been under continuous growth monitoring for almost 4 decades.

## Methods

### Study design and participants

We did a retrospective cohort study using routine growth monitoring data for all children whose date of birth had been recorded to assess trends in growth faltering in children younger than 2 years in the West Kiang region of The Gambia during the past four decades.

Three rural villages in this region (Keneba, Manduar, and Kantong Kunda) have benefited from free health care provided by the UK Medical Research Council for the past 40 years. Since the 1970s there have been increasing levels of support and interventions (panel) such that these villages have benefited from unprecedented levels of nutrition-specific and nutrition-sensitive interventions compared with other such communities in rural low-income settings. Growth monitoring was done on a monthly basis in the 1970s but from 1983 onward, measurements were made at birth, 6 weeks, 3 months and then every 3 months thereafter. Diseases were recorded both at regular child ‘well baby’ clinics and when mothers presented with a sick child, and here we focus on clinical diagnoses for pneumonia, chest infections, diarrhoea, and malaria. Malaria diagnoses were based on positive blood films and, since 2007, on rapid diagnostic tests.^9^

As described elsewhere,^9^ the climate in the intervention area has a long, dry harvest season (November to May) and a wet so-called ‘hungry’ season (late June to mid-October) when agricultural work, depletion of food supply, and infectious diseases are at their peak.

Ethics approval for the demographic surveillance of the three villages was granted by the Joint Gambian Government/Medical Research Council Unit The Gambia Ethics Committee.

### Procedures

Standard anthropometric measurements were done in the clinic by trained clinic staff and *Z* scores were calculated against the WHO 2006 growth standards.^10^ We defined stunting, wasting, and underweight as height-for-age, weight-for-length, and weight-for-age of less than 2 SDs (*Z* scores) below the WHO reference median. Further details are provided in the appendix.

### Statistical analysis

We fitted the effects of age and season on repeated growth parameters using random effects models. Models for boys and girls and each decade were fitted separately. To describe secular changes in rates of stunting, wasting, and underweight, we fitted random effects logistic regression of the binary variable on the first four orthogonal polynomials in age and the first pair of Fourier terms for season (appendix). To describe the effect of season on growth, we obtained seasonal patterns of body size by Fourier regression, as described in the appendix.^11^ To describe the changes in body size with age, we produced plots of mean *Z* score versus age by fitting age with ten-knot cubic regression splines and controlling for season by including the first pair of Fourier terms. We quantified growth faltering as the drop in *Z* score during the 18 month interval between 3 months and 21 months of age. These estimates are all simple linear combinations of the regression coefficients and their standard errors calculated using the variance–covariance matrix for the regression coefficients (ie, the Fisher information matrix), using Stata's post-estimation command *lincom*. We did not do any formal statistical hypothesis tests. With such large volumes of observational data almost any difference examined would be significant, so statistical significances poorly discriminate between important and trivial patterns in the data. Instead, we focused on estimation of effect sizes and their confidence intervals. All analyses were done with Stata 12.

### Role of the funding source

The UK Medical Research Council has provided sustained support for our unique cohort over many years and approved our general research plans (including longitudinal data collection) every 5 years. MRC played no other role in interpretation of the data or preparation of the manuscript. The corresponding author had access to all of the data and had final responsibility for the decision to submit for publication.

## Results

From May 1, 1976, to Feb 29, 2012, 4474 children younger than 2 years from these villages were seen at the child clinics in Keneba. Children were included in this analysis if their date of birth was known accurately and they visited the clinic on six or more occasions, giving a total of 3659 children eligible for the study. Those ineligible included 24 with unknown date of birth and 791 visitors who attended the clinic on five or fewer occasions. The median number of visits per child was 16 (IQR 13–26), resulting in a total of 59 371 visits at which anthropometric measurements were made. Most deliveries occurred at home in the presence of a traditional birth attendant but a trained midwife completed a baby check including anthropometric measurements within 5 days of delivery (mostly within 72 h).

**Figure 1:**
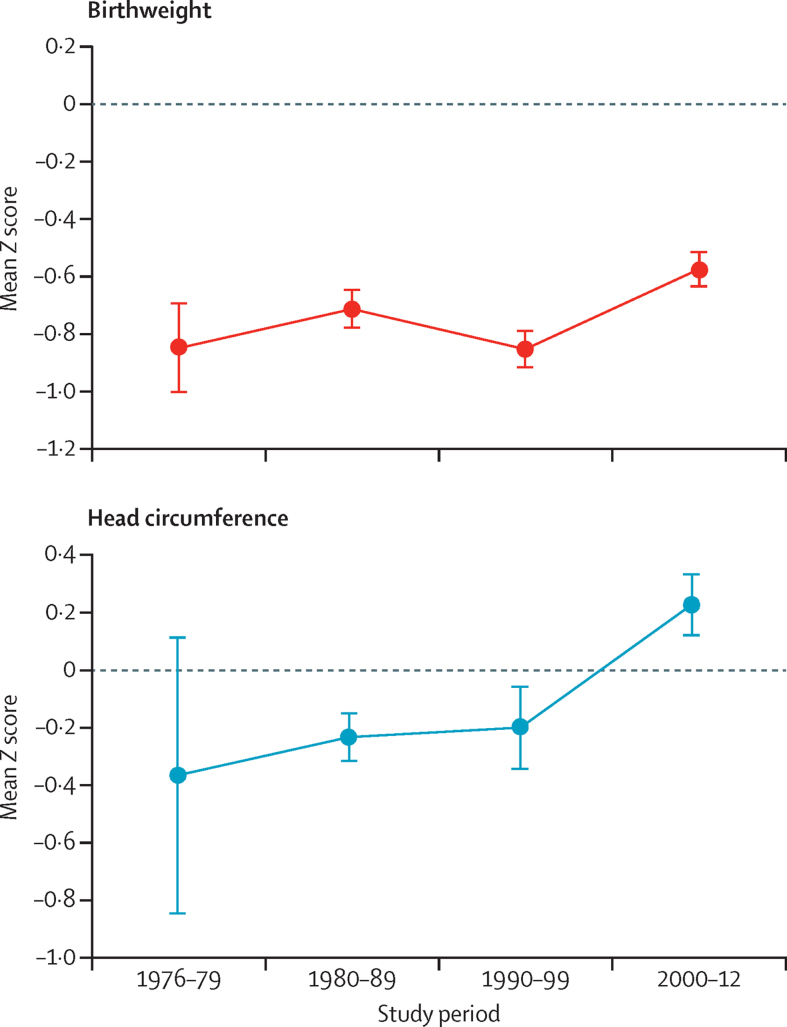
Secular changes in weight and head circumference at birth Figures shows mean (SE) *Z* scores for both sexes combined, calculated using the WHO 2006 growth reference standards.

We analysed secular trends in birth size, because it is an important determinant of postnatal growth and attained size. Data about birth size were available for 2728 (75%) babies (figure 1, table 1). We excluded length data because there were more missing data than for weight and head circumference and because birth lengths measured with a length mat in the babies' homes are inherently less reliable than the other measurements. During the four decades of the study period, birthweight Z score increased by 0·26 (95% CI 0·18 to 0·34) from a starting point of −0·85 (figure 1, table 2). Head circumference at birth Z score increased by 0·58 (95% CI 0·33 to 0·83) from −0·36, thus ending up slightly above the WHO standards at 0·22 (95% CI 0·11 to 0·33). A small part of this increase might be attributable to a steady increase in maternal height totalling 28 mm (95% CI 18 to 38; table 1).

**Figure 2:**
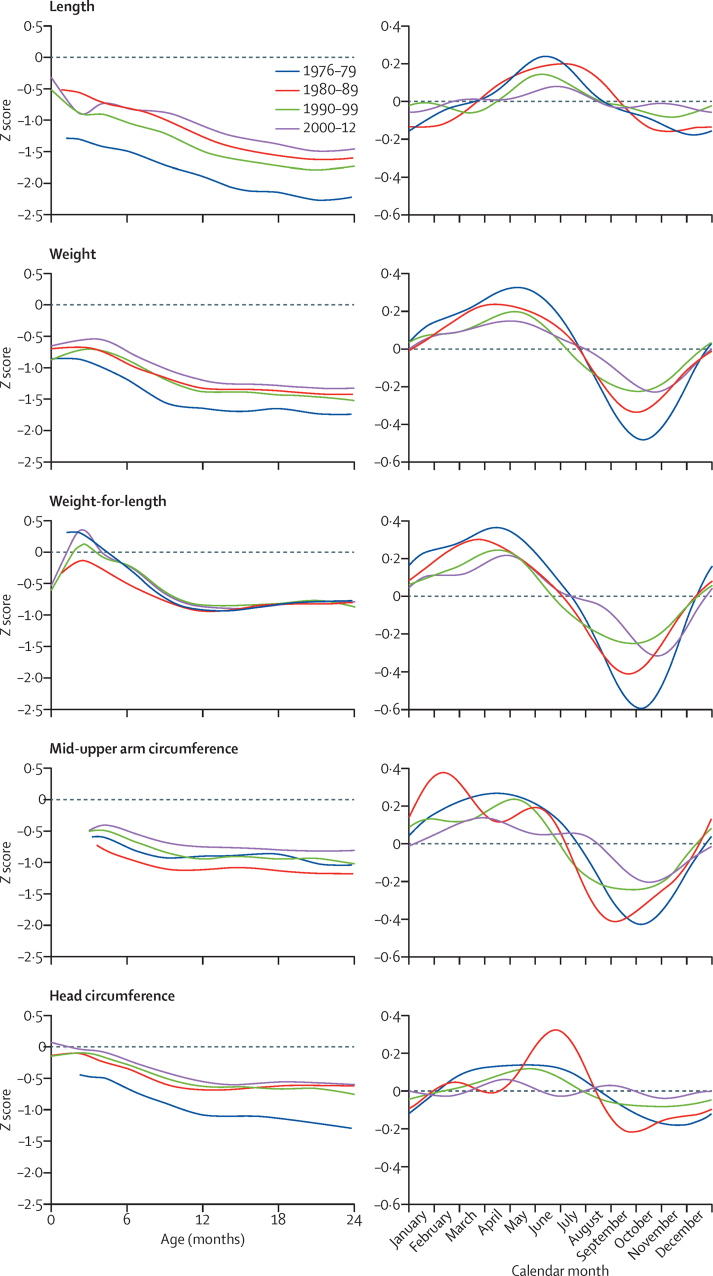
Secular and seasonal trends in child growth Figure shows mean age and *Z* scores for sexes combined, calculated by comparison with the WHO 2006 growth reference standards. Length refers to length-for-age; weight refers to weight-for-age.

Figure 2 captures the characteristic growth patterns of these rural infants. They are born small and continue to fall away from the WHO standard length centiles throughout the first 2 years of life. Their weight shows early catchup while the infants are still fully breastfed and largely protected from infections; this trend is magnified in their weight-for-length due to the simultaneous decline in length. Mid-upper-arm and head circumferences show a similar resilience in very early infancy.

The figure also illustrates the secular trends in growth during the four decades. Length shows a consistent, but limited, improvement. At 2 years, length-for-age *Z* score had improved by 0·74 (95% CI 0·59 to 0·89) from a starting point of −2·10 (table 2). Weight and head circumference showed an initial improvement by the second decade but little further gain. Weight-for-length showed absolutely no change in the second year of life. Mid-upper-arm circumference increased by a quarter of a *Z* score (table 2).

**Figure 3:**
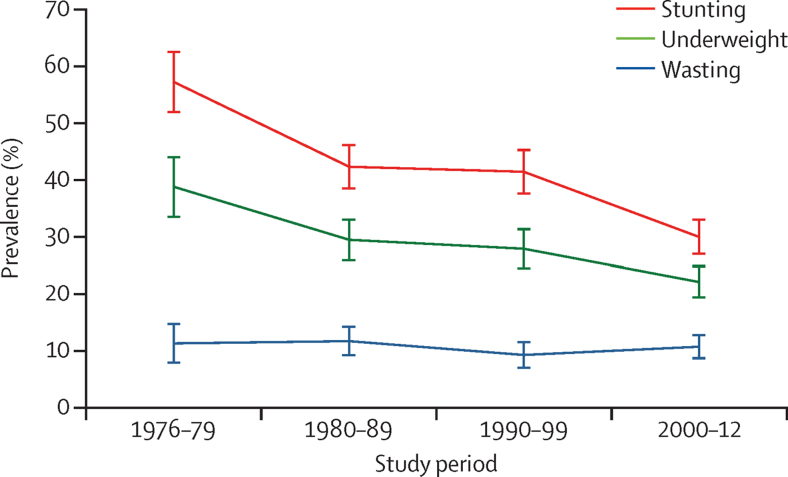
Secular trends in stunting, underweight, and wasting at 2 years of age Stunting, underweight, and wasting are defined as proportion below −2 *Z* scores against WHO 2006 growth reference standards.

The prevalence of stunting at 2 years almost halved from 57% to 30% and the prevalence of underweight decreased from 39% to 22% (table 2, figure 3). There was no change in the prevalence of wasting.

**Figure 4:**
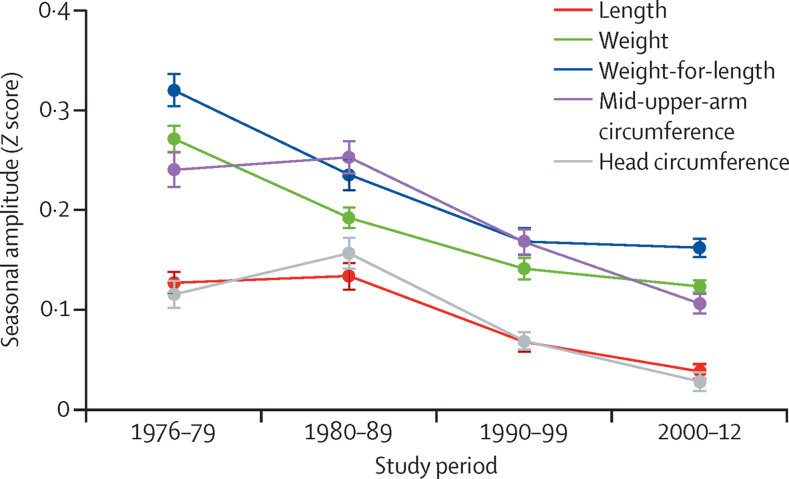
Amplitude of the seasonality by decade Figure shows seasonal *Z* score amplitude for sexes combined, calculated by comparison with the WHO 2006 growth standards.

Growth failure is markedly seasonal in this environment (figure 2), with greater deficits occurring in the rainy season (July to November) when infections are more common and maternal care declines due to the pressures of farming. Figure 4 shows that there has been a substantial attenuation of the seasonality of growth during the four decades studied. When assessed as the amplitude of *Z* score fluctuation, this measure was significant for all indices (table 2) in the order of a tenth of a *Z* score.

**Figure 5:**
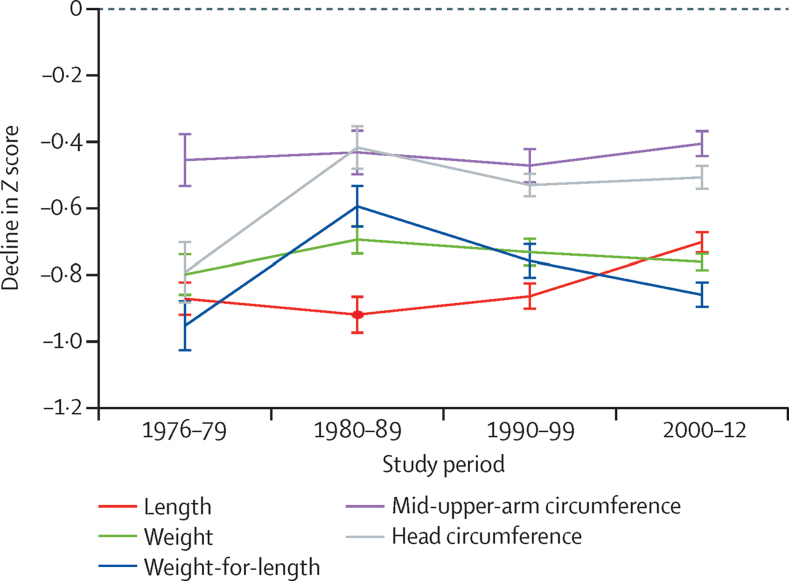
Fall in *Z* scores between 3 and 21 months of age for each decade Figure shows fall in *Z* scores for sexes combined, calculated by comparison with the WHO 2006 growth standards.

We defined growth faltering on the basis of the differences in *Z* score between 3 months and 21 months post partum. In the 1970s, *Z* scores for length-for-age, weight-for-age, weight-for-length, and head circumference all fell by between 0·79 and 0·95 (table 2). Over time, this fall was slightly attenuated for length-for-age (*Z* score 0·17, 95% CI 0·11–0·23) and weight-for-length (0·09, 0·01–0·17), and more markedly attenuated for head circumference (0·28, 0·18–0·38; figure 5). The decline in *Z* score for weight-for-age and mid-upper-arm circumference did not change during the period studied.

**Figure 6:**
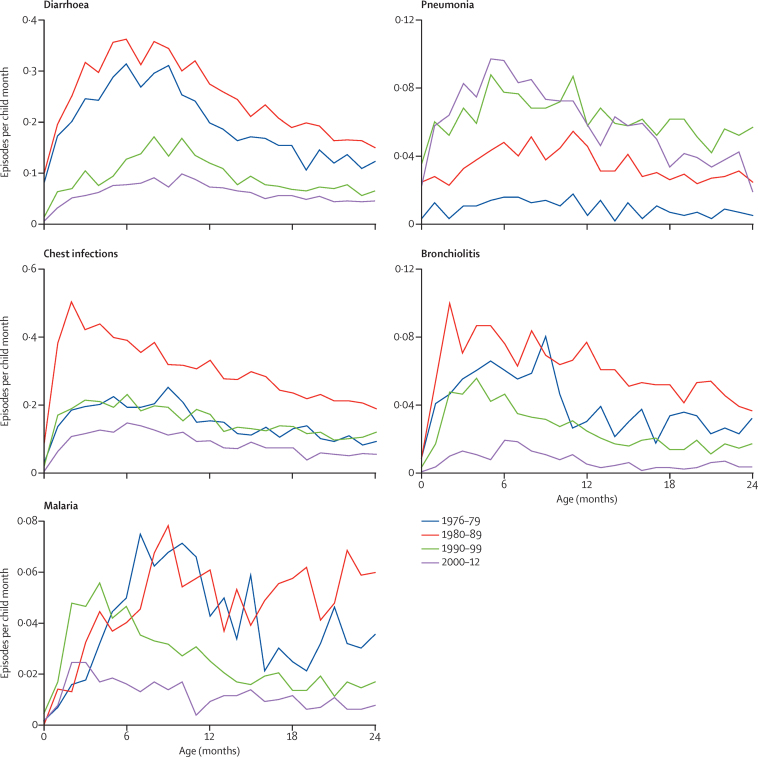
Disease episodes for each decade Figure shows episodes per child per month between 0 months and 24 months of age. The apparent contrary increase in the incidence of pneumonia probably results from the changing clinical definitions of pneumonia among the series of doctors who have worked in the Medical Research Council Keneba clinic during the past four decades, especially with the introduction of WHO Integrated Management of Childhood Illness guidelines in 1997.^20^ It is also likely that, with the more stringent WHO definitions for malaria, pneumonia was frequently stated as the diagnosis. This phenomenon has been reported in other settings in the tropics.^21^

The incidence of diarrhoea, malaria, and bronchiolitis in the children younger than 12 months fell by 80% during the four decades studied. Conversely, the incidence of pneumonia seemed to increase during the four decades (figure 6).

## Discussion

Goal 2 of the SDGs, “to end hunger, achieve food security and improved nutrition, and promote sustainable agriculture”, is accompanied by the target to achieve the internationally agreed goals for stunting and wasting in children younger than 5 years by 2025. For stunting, this goal would require a 40% reduction from the current estimate of 159 million stunted children to reach the target of less than 100 million. In Africa there has been a disappointing decline in the prevalence of stunting from 42% in 1990 to 32% in 2015^12^ and, because of population growth, the absolute numbers of children with stunting actually increased from 47 million to 58 million during this period. The prevalence of stunting is now predicted to stabilise at that level because continued population growth offsets a slower-than-required decline in prevalence. By comparison, during the same period the prevalence of stunting in Asia decreased from 48% to 25% and the total number of children with stunting declined from 189 million to 84 million.^12^

Elimination of stunting creates a complex and paradoxical challenge, which suggests that one or more key causative factors remain unknown. On the one hand, nutrition-specific interventions have repeatedly shown very limited efficacy even when implemented under the optimal conditions of randomised trials,^2^, ^13^, ^14^, ^15^, ^16^, ^17^ whereas on the other hand, stunting resolves rapidly as wealth and living conditions improve in countries passing through the economic transition.^11^, ^18^

The longitudinal data presented in this study add to this challenge. During almost four decades the Medical Research Council has made sustained investments in health care and nutrition-related infrastructure within our core study villages; these inputs are unparalleled across rural Africa and would be prohibitively expensive for governments of low-income countries to roll out nationwide. These villages have access to antenatal and postnatal care, and round-the-clock access to clinicians and nurses in a well equipped and efficient primary health-care clinic. All health services are free of charge. All children are fully vaccinated, receive vitamin A, mebendazole, and other health interventions as per WHO protocols. Breastfeeding rates are among the very best worldwide and are further supported by Baby Friendly Community Initiatives accompanied by regular messaging in support of exclusive breastfeeding for 6 months. Open defecation and water obtained from contaminated open wells have been universally replaced by latrines in all compounds and tube well water supplied through clean pipes to standpipes around the villages. These interventions have had a profound effect on mortality in children younger than 5 years^9^ and the incidence of most diseases, especially diarrhoea (which has been previously implicated as a major cause of growth failure; figure 6).^19^ Further, children attend regular well-baby checks with growth monitoring and we provide a dedicated treatment centre for severely malnourished children to treat those who do become malnourished. The remittance economy from village members who have migrated overseas, together with incomes from employment at the Medical Research Council, have greatly improved food security and attenuated the stress of the so-called hungry season as reflected in the reduction in the amplitude of seasonal growth faltering in figure 4. This increased wealth has also improved housing conditions and dispersed families over a wider area, reducing overcrowding. Child mortality has fallen, birth spacing has increased, and family size has decreased. There is now free universal primary education with enrolment of about 97%, although this figure drops for secondary education particularly for girls to 30%.^22^ Furthermore we have, over the years, conducted and published a series of randomised trials of nutritional interventions targeted at pregnant and lactating mothers, infants, and children, with the main aim to improve growth (appendix). Our findings have shown at most modest improvements in infant growth, consistent with results from systematic reviews and meta-analyses.^2^, ^13^, ^23^

The modest increase of 2·8 cm in the mean maternal height is indicative of a small degree of improvement in maternal nutrition during the four decades. Meta-analysis of the relationship between maternal height and birthweight^24^ yielded an expected effect of 8 g more birthweight per cm of maternal height. The COHORTS group reported a similar value of 0·024 *Z* scores per cm.^25^ Therefore the increase in maternal height probably contributed only about 20–30 g of the observed 120 g increase in birthweight.

A limitation for our data was the difficulty in deriving a consistent sampling frame for the population under study in an area undergoing rapid change, particularly in the later decades. Changes in the population structure during the past 40 years might have influenced the trends we have reported. We attempted to control for this factor by excluding the children who attended our clinic fewer than six times as likely visitors. However, exclusion of these children might have created a sampling bias, because infrequent attenders might represent resident children who engaged poorly with health care. This potential bias would only affect the trends displayed if the population prevalence of poor attenders changed during the period studied.

Another limitation was missing data from the 1970s, particularly birth data, limiting our ability to evaluate the trends in these parameters. Additionally, we omitted birth length data because of poor reliability in the measurements and the small number of measurements that were available in all the four decades. Comparison of the trends noted in our core study villages with those in neighbouring villages receiving less intensive intervention would have been desirable, but such data were not available.

Growth has improved during these four decades but, despite the unprecedented levels of investment, the prevalence of low birthweight (12%), childhood stunting (30%), and underweight (22%) remains high. The prevalence of wasting has not changed, and growth faltering between 3 months and 21 months has been only marginally attenuated. These data suggest that the refractory stunting must be caused by factors (beyond the improvements and interventions provided in the study villages) that are corrected as nations pass through the economic transition and advance from low-income and lower-middle-income status. Environmental enteropathy affecting almost all children in low-income settings has been proposed as the mechanism linking growth failure with WASH deficits.^26^ Our results, together with a previous analysis^27^ of associations between poor child growth and a range of indicators of socioeconomic status and living conditions in this same community, suggest that there is a very high threshold for WASH improvements that must be achieved before growth faltering can be eliminated. Improved housing conditions, possibly including the provision of piped water directly into the home, might be a necessary step in the global challenge to eliminate childhood malnutrition.

Our study villages of Keneba, Kantong Kunda, and Manduar are highly unusual (and possibly unique) in having the combination of intensive interventions over a protracted period accompanied by systematic growth monitoring; our results might therefore not be generalisable. However, before Medical Research Council inputs and in all other respects such as environment and farming practices they share many characteristics with countless other rural villages in sub-Saharan African in areas of low malaria endemicity. Therefore, we believe that our findings and suggestions for future interventions are likely to be applicable to other similar settings in rural Africa.

## Figures and Tables

**Table 1:** Maternal and infant baseline data

		**1976–79 (n=560)**	**1980–89 (n=920)**	**1990–99 (n=879)**	**2000–12 (n=1300)**	**Overall (n=3659)**
Sex						
	Boys	267	453	455	665	1840
	Girls	293	467	424	635	1819
Mean (SD) birthweight, kg (n=2728)						
	Boys	3·1 (0·3)	3·1 (0·4)	3·0 (0·4)	3·1 (0·4)	3·0 (0·4)
	Girls	2·8 (0·5)	2·9 (0·4)	2·8 (0·4)	3·0 (0·4)	2·9 (0·4)
Premature		15/76 (20%)	58/714 (8%)	63/721 (9%)	84/687 (12%)	220/2198 (10%)
Primiparity		55/478 (12%)	94/867 (11%)	79/788 (10%)	175/1176 (15%)	403/3300 (12%)
Mean (95% CI) maternal height, cm		158·1 (157·4–158·9)	158·4 (157·7–159·1)	159·4 (158·8–160·0)	160·9 (160·3–161·6)	159·8 (150·6–170·4)

Data are mean (SD), n/N (%), or mean (95% CI).

**Table 2:** Effect size estimates for changes in body size by decade

		**1970s**	**2000s**	**Change between 1970s and 2000s**
Birth (mean WHO *Z* scores)				
	Birthweight	−0·85 (−1·00 to −0·70)	−0·59 (−0·65 to −0·53)	0·26 (0·18 to 0·34)
	Head circumference	−0·36 (−0·84 to 0·12)	0·22 (0·11 to 0·33)	0·58 (0·33 to 0·83)
Sex difference at birth (male minus female mean WHO *Z* scores)				
	Birthweight	0·47 (0·18 to 0·76)	−0·03 (−0·15 to 0·09)	−0·50 (−0·81 to −0·19)
	Head circumference	0·82 (−0·20 to 1·84)	−0·05 (−0·26 to 0·16)	−0·87 (−1·91 to 0·17)
Body size at 2 years (mean WHO *Z* scores)				
	Length-for-age	−2·10 (−2·22 to −1·98)	−1·36 (−1·44 to −1·27)	0·74 (0·59 to 0·89)
	Weight-for-age	−1·64 (−1·77 to −1·51)	−1·20 (−1·28 to −1·11)	0·44 (0·29 to 0·59)
	Weight-for-length	−0·71 (−0·82 to −0·59)	−0·68 (−0·76 to −0·60)	0·03 (−0·11 to 0·17)
	Mid-upper-arm circumference	−1·02 (−1·14 to −0·89)	−0·74 (−0·82 to −0·66)	0·28 (0·13 to 0·43)
	Head circumference	−1·28 (−1·48 to −1·08)	−0·51 (−0·59 to −0·43)	0·77 (0·55 to 0·99)
Prevalence of stunting, underweight, and wasting at 2 years				
	Stunting	57·1% (51·9 to 62·4)	30·0% (27·0 to 33·0)	−27·1% (−33·2 to −31·5)
	Underweight	38·7% (33·5 to 44·0)	22·1% (19·4 to 24·8)	−16·6% (−22·5 −21·1)
	Wasting	11·3% (7·9 to 14·8)	10·8% (8·7 to 12·8)	−0·5% (−4·5 to −3·2)
Growth faltering*				
	Length-for-age	−0·87 (−0·92 to −0·82)	−0·70 (−0·73 to −0·67)	0·17 (0·11 to 0·23)
	Weight-for-age	−0·80 (−0·86 to −0·74)	−0·76 (−0·78 to −0·74)	0·04 (−0·03 to 0·11)
	Weight-for-length	−0·95 (−1·02 to −0·88)	−0·86 (−0·89 to −0·82)	0·09 (0·01 to 0·17)
	Mid-upper-arm circumference	−0·45 (−0·53 to −0·38)	−0·40 (−0·44 to −0·37)	0·05 (−0·04 to 0·14)
	Head circumference	−0·79 (−0·88 to −0·70)	−0·51 (−0·54 to −0·47)	0·28 (0·18 to 0·38)
Seasonality†				
	Length-for-age	0·13 (0·12 to 0·14)	0·04 (0·03 to 0·05)	−0·09 (−0·10 to −0·08)
	Weight-for-age	0·27 (0·26 to 0·28)	0·12 (0·12 to 0·13)	−0·15 (−0·16 to −0·14)
	Weight-for-length	0·32 (0·30 to 0·34)	0·16 (0·15 to 0·17)	−0·16 (−0·18 to −0·14)
	Mid-upper-arm circumference	0·24 (0·22 to 0·26)	0·11 (0·10 to 0·12)	−0·13 (−0·15 to −0·11)
	Head circumference	0·12 (0·10 to 0·13)	0·03 (0·02 to 0·04)	−0·09 (−0·11 to −0·07)
	Annual incidence of diarrhoeal infection between birth and age 2 years‡	3·13 (2·89 to 3·37)	0·84 (0·76 to 0·91)	−2·29 (−2·54 to −2·04)

Data are effect size (95% CI).

* Difference in mean *Z* score between 3 months and 21 months.

† Amplitude of seasonal *Z* score fluctuation between birth and age 2 years.

‡ Diarrhoea is the infection usually associated with growth faltering.

## Panel Timeline of interventions introduced for three rural Gambian villages

**1976–79**

• Lower basic primary education (grades 1–4)
• Routine daily food supplementation for all infants aged 3–12 months
• Routine child health clinic
• Infant vaccines (BCG, measles, yellow fever, oral polio, combined diptheria, tetanus, pertussis), maternal vaccine (tetanus toxoid)
• Antenatal clinic
• Free availability of oral rehydration solution
• Health and nutrition education

**1980–89**

• International Trypanotolerance Centre provided hand pumps and tap water
• Pit latrines in compounds
• Medical Research Council Gate clinic opened
• Mobile midwifery service
• Infant vaccine (hepatitis B)

**1990–99**

• Upper basic primary education
• Emigration of young people (education and remittance)
• Vitamin A supplementation in infants
• Infant vaccine (*Haemophilus influenzae* type B)

**2000–12**

• Infant vaccines (pneumococcal and rotavirus)
