# Supplementary material for: Growth faltering in rural Gambian children after four decades of interventions: a retrospective cohort study
Source: Lancet Glob Health. 2017 Jan 17;5(2):e208–16. doi: 10.1016/S2214-109X(16)30355-2 (PMC5340725; doi:10.1016/S2214-109X(16)30355-2)
Supplement: Supplementary appendix [file mmc1.pdf]

THE LANCET Global Health

Supplementary appendix

This appendix formed part of the original submission and has been peer reviewed. We post it as supplied by the authors.

Supplement to: Nabwera HM, Fulford AJ, Moore SE, Prentice AM. Growth faltering in rural Gambian children after four decades of interventions: a retrospective cohort study. *Lancet Glob Health* 2017; **5:** e208–16.

Growth faltering in rural Gambian children after four decades of interventions: a retrospective cohort study

Helen M Nabwera BM BS, [1, 2] Anthony J Fulford PhD, [1, 2] Sophie E Moore PhD, [1, 3] Andrew M Prentice PhD [1, 2]

1. MRC Unit, The Gambia (HMN, AJF, SEM, AMP)
2. MRC International Nutrition Group, London School of Hygiene and Tropical Medicine, London, UK (HMN, SEM, AJF, AMP)
3. Division of Women's Health, King's College London, London, UK (SEM)

Supplementary material- contents

1. Supplementary study population
2. Supplementary methods
3. Supplementary tables (2)
4. Supplementary references

1. Supplementary study population

The UK Medical Research Council has supported a research centre in the remote rural Gambian village of Keneba since 1949. Studies initially consisted of demographic records of births, deaths, marriages and migrations collected by village recorders and an annual survey of health and nutritional status in Keneba and 3 nearby villages: Manduar, Kantong Kunda and Jali.[1] In 1974 the Dunn Nutrition Unit commenced more intensive research and provided full-time clinical services to 3 of the villages (Jali declined to participate).

2. Supplementary methods

Anthropometry

The anthropometry measurements were performed in the clinic by trained clinic staff. Weight measurements were performed with the infants unclothed and recorded in kilograms to two decimal places. In the early decades, birth weights and subsequent weights were recorded using manual Salter spring balance and tared sling (Salter Industrial Measurements Ltd, West Bromwich, United Kingdom) and Todd Scales, Cambridge, UK. In the later decades electronic Seca 336 high precision portable baby weighing scales were used. All the weighing scales were calibrated regularly. Lengths were measured on Holtain infantometer (Holtain, Crymmych, UK) in the early decades but more recently on Kiddimetre (Raven Equipment, Great Dunmow, Essex, UK). The Leicester plastic stadiometer was used to measure maternal heights in the later years. Tape measures were used to measure the infants' mid upper arm circumferences and head circumferences. Sex and age-adjusted z-scores for weight for age (WAZ), length for age (LAZ), weight for length (WLZ), mid upper arm circumference (MUACZ), head circumference (HCZ) and birth weight (BWTZ) were calculated by comparison to the WHO 2006 growth standards.[2] Stunting, wasting and underweight are defined respectively as height-for-age, weight-for-length, weight-for-age of 2 or more standard deviations below the WHO reference median.

We define growth faltering in terms of the fall in z-scores between 3 and 21 months in order to avoid both the complications of catch-up growth in the first 3 months and the inevitably poorer estimates of the curves at the extremes of the data range.

Statistical Methods

Effects of age and season on repeated growth parameters were fitted using random effects models. Models for males and females and each decade were fitted separately. To describe secular changes in growth by decade i.e. rates of stunting, wasting and underweight in 2 year olds plotted in Figure 4, we fitted random effects logistic regression of

the binary variable on the first four orthogonal polynomials in age and the first pair of Fourier terms for season (see below).

To describe the effect of season on growth, seasonal patterns of body size were obtained by "Fourier regression". [3,4] Briefly, Fourier regression represents the seasonal pattern as a Fourier series whose higher order terms are regarded as high-frequency noise and discarded. The resulting truncated series is a linear combination of trigonometric functions of θ, the angle representing the phase of the year when the measurement was made, and whose coefficients are readily estimated as part of the regression model. We fitted the first three pairs of Fourier terms and controlled for age by including the first three orthogonal polynomials in age (*age*1, *age*2, *age*3) in the model. Thus the jth observation for the ith individual is given by:

$$Y_{ij} = \beta_0 + \beta_1 age1_{ij} + \beta_2 age2_{ij} + \beta_3 age3_{ij} + \sum_{k=1}^{3}\left[\alpha_k \sin\left(k\theta_{ij}\right) + \beta_k \cos\left(k\theta_{ij}\right)\right] + \tau_i + \varepsilon_{ij}$$

where the α_k and γ_k are the coefficients for the Fourier terms and the βs the remaining regression coefficients; τ_i is the random effect due to the ith individual and ε_{ij} is the error term. We appreciate that seasonal patterns may themselves vary with age. Allowing for this would have unduly complicated the analysis and is unnecessary for the purpose of this paper. The seasonal patterns we estimate are therefore averaged across all age groups.

In order to quantify the children's susceptibility to seasonal changes (as plotted in Figure 5) we estimated the amplitude of the seasonal pattern, which we define as the square root of half the sum of the squared Fourier coefficients:

$amplitude = \sqrt{\sum_k [\alpha_k^2 + \gamma_k^2]\,/2}$.[5]

The delta method was used to estimate the standard error of these estimates (employing Stata's post-estimation command *nlcom*).

To describe the changes in body size with age, plots of mean z-score versus age (Figure 2) were produced by fitting age with 10-knot cubic regression splines and controlling for season by including the first pair of Fourier terms.

Estimates of mean values and their standard errors calculated at particular ages for each sex and decade were taken from the predicted values yielded by the above regression models. We quantify growth faltering as the drop in z-score over the 18-month interval starting at 3 months of age. These estimates are all simple linear combinations of the regression coefficients and their standard errors calculated using the variance-covariance matrix for the regression coefficients i.e. the Fisher information matrix (employing Stata's post-estimation command *lincom*).

The estimation of age specific disease incidence over the different decades was done by dividing the total number of children diagnosed with the disease in each age group as numerator, by total number of children 2 years of age or under who were seen in the clinic.

We perform no formal statistical hypothesis tests. With such large volumes of observational data almost any difference examined would be significant so statistical significances provide poor means of discriminating between important and trivial patterns in the data. Instead we focus on estimating effect sizes and their confidence intervals. All analysis was performed using Stata 12. (StataCorp, College Station,TX).

3. Supplementary tables

Table 1: Summary of previous studies that have assessed trends in growth faltering in African children

Study	Study design (interval)	Country	N (age group)	Main findings
**Said-Mohammed et al. 2015**[6]	Systematic review (1970-2013)	South Africa	50 studies (under 6 years)	NCHS reference, from 1993 to 2003 the prevalence of stunting increased by 2.9 % (z-test, p < 0.05). WHO standard, the prevalence of stunting decreased by 5.9 % between 1999 and 2013 (z-test, p < 0.001). However, the 2008 National Income Dynamic Study (NIDS) showed an increase of 6.8 % from the 2005 National Food Consumption Survey (z-test, p < 0.001).
**Gray S et al. 2010**[7]	Longitudinal study (1998-2004)	Uganda	123	Noticeable declines in weight velocity occurred in the fourth month and after the sixth month. Weight gain was static after the second year, when upward of 40% of children were clinically underweight.
**Kalanda BF et al. 2005**[8]	Longitudinal study	Malawi	(0-1 y)	Low birthweight infants were shorter and lighter throughout infancy than either normal birthweight or international reference values.<br><br>At 12 months, placental or peripheral malaria at delivery (adjusted odds 1.8; 1.0, 3.1), number of infant illness episodes (AOR = 2.1; 1.2, 3.6) and maternal illiteracy (AOR = 2.7; 1.5, 4.9) were independently associated with low weight for age.<br><br>Maternal short stature (AOR = 1.8; 1.1. 3.2), male sex (AOR = 2.4; 1.4, 4.1), number of infant illness episodes (AOR = 2.6; 1.5, 4.4), and birth in the rainy season (2.1; 1.2,

				3.7) were independently associated with stunting.<br><br>Placental or peripheral malaria at delivery (AOR = 2.2; 1.1, 4.4) and number of illness episodes (AOR = 2.2; 1.1, 4.5) were independently associated with thinness.
**Hauspie RC et al 1989**[9]	Longitudinal study	Democratic Republic of Congo	4030<br><br>(0-4 y)	Growth weight velocity slows down below average in the rainy seasons.

Table 3: Summary of randomised trials of nutrition interventions in The Gambia aimed at improving growth

Study	Study design	Country	N<br><br>(mother/ infant/children or mother-infant pairs)	Main findings
**Johnson et al. 2016**[10]	RCT	The Gambia	620	Despite evidence of between-arm differences in some fetal biometry, z-scores at birth were not greater in the intervention arms than the FeFol arm (e.g., birth weight z-scores: FeFol -0.71, MMN -0.63, PE -0.64, PE + MMN -0.62; group-wise p = .796). In regression analyses, intervention associations with birth weight and head circumference were modified by maternal weight gain between booking and 30 weeks gestation (e.g., PE + MMN associations with birth weight were +0.462 z-scores (95% CI [0.097, 0.826]) in the highest quartile of weight gain but -0.099 z-scores (-0.459, 0.260) in the lowest).
**Goldberg et al. 2013**[11]	RCT	The Gambia	525	No significant effect of calcium supplementation on infant growth.<br><br>(Not designed with birth weight as an outcome therefore, it wasn't powered to look at birthweight.)
**van de Merwe et al. 2013**[12]	RCT	The Gambia	172	PUFA supplementation resulted in a significant increase in plasma n-3 LC-PUFA concentrations (P < 0.001 for both DHA and EPA) and mid upper arm circumference (MUAC) (effect size: 0.31 z scores; 95% CI: 0.06, 0.56; P = 0.017) at 9 mo of age. At 12 mo, MUAC remained greater in the intervention group, and we observed

				significant increases in skinfold thicknesses (P ≤ 0.022 for all). No other significant differences between treatment groups were detected for growth or LMRs at 9 mo or for secondary outcomes.
**Unger S [13] 2013 (Thesis)**	RCT	The Gambia	1101	Multiple micronutrient supplementation was associated with a small increase in height-for-age z-scores 24wk after recruitment (effect size for MMN groups combined: 0·084 SD/24wk, 95%CI: 0·005, 0·168; p=0·037; equivalent to 2-5mm depending on age).
**Williams et al. 2007[14]**	RCT	The Gambia	93	Gambian infants showed a seasonal deterioration in growth and persistently elevated acute phase protein concentrations and intestinal permeability. Oral supplementation with glutamine did not improve growth (x +/- SE: weight gain, 60 +/- 19 and 69 +/- 20 g/mo; length gain, 1.01 +/- 0.05 and 0.95 +/- 0.03 cm/mo) or intestinal permeability [lactulose:mannitol ratio: 0.29 (95% CI: 0.23, 0.35) and 0.26 (95% CI: 0.21, 0.32)] in the glutamine and placebo groups, respectively.
**Darboe et al. 2007[15]**	RCT	The Gambia	197	Apart from a transient diff erence in length between the treatment groups at 6 months (in favour of WHO dose of Vitamin A), there was no detectable effect of the supplementation regimen on growth.
**Jarjou et al. 2006[16]**	RCT	The Gambia	125	No significant differences were detected between the groups in breast-milk calcium concentration, infant birth weight, or growth or bone mineral status during the first year of life.
**Krähenbühl et al. 1998[17]**	RCT	The Gambia	90	Neither the high fat nor the high carbohydrate supplement had an effect on weight or height gain. The high fat supplement did slightly increase adipose tissue mass.
**Ceesay et al. 1997[18]**	RCT	The Gambia	2047	Maternal weight gain increased by 201 g (P < 0.001) in the hungry season, by 94 g (P < 0.01) in the harvest season (November to May), and by 136 g (P < 0.001) over the whole year. The odds ratio for low birthweight babies in supplemented women was 0.61 (95% CI 0.47 to 0.79, P < 0.001). Head circumference was significantly increased (P < 0.01), but by only 3.1 mm. Birth length and duration of gestation were not affected.
**Hoare S et al. 1996[19]**	RT	The Gambia	40	With a 50% increase in energy intake and a 100% increase in protein intake there was a

				rapid and highly significant (P < 0.001) gain in weight within a fortnight whether the supplement was given immediately or 2 weeks after presentation. Rates of weight increase were similar whether supplementation was provided early or late, but over the full 28 d (of intervention and non-intervention) children who received late supplementation had greater overall weight gain (P < 0.02) than those supplemented early.
**Bates et al. 1993[20]**	RCT	The Gambia	110	Body weights and arm circumferences showed a linear increase, plus a seasonal effect (rainy season faltering). For body weight there was no significant overall effect of the supplement. For arm circumference, a very small (2%) but significant (P < 0.01) difference favoured the supplemented group.
**Prentice AM et al. 1987[21]**	RCT	The Gambia	379	Supplementation was ineffective during the dry season but highly effective during the wet season: +225 +or- 56 grams, p=0.001 (unadjusted) or +200 +or- 53 grams, p=0.001 (adjusted for sex, season, and parity) by between-child multiple regression analysis; +231 +or- 65 grams, p=0.001 by within-mother analysis. The proportion of babies of low birthweight (2501 grams) decreased from 23.7 to 7.5%, p=0.002.

4. Supplementary references

1. Hennig BJ, Unger SA, Dondeh BL, et al. Cohort Profile: The Kiang West Longitudinal Population Study (KWLPS)-a platform for integrated research and health care provision in rural Gambia. *International journal of epidemiology.* 2015. doi: 10.1093/ije/dyv206

2. World Health Organisation. The WHO Growth Reference Standards. 2006; http://www.who.int/childgrowth/en/. Accessed 14/11/2015.

3. United Nations. Sustainable Development Goals. 2015; https://sustainabledevelopment.un.org/?menu=1300. Accessed 10/10/2015.

4. Rayco-Solon P, Moore SE, Fulford AJ, Prentice AM. Fifty-year mortality trends in three rural African villages. *Tropical Medicine & International Health.* 2004; **9**: 1151-1160.

5. Fulford AJ. The coefficient of cyclic variation: a novel statistic to measure the magnitude of cyclic variation. *Emerging Themes in Epidemiology.* 2014; **11**: 15.

6. Said-Mohamed R, Micklesfield LK, Pettifor JM, Norris SA. Has the prevalence of stunting in South African children changed in 40 years? A systematic review. *BMC public health.* 2015; **15**: 534.

7. Gray S, Akol HA, Sundal M. Longitudinal weight gain of immunized infants and toddlers in Moroto District, Uganda (Karamoja subregion). *American Journal of Human Biology.* 2010; **22**: 111-123.
8. Kalanda BF, van Buuren S, Verhoeff FH, Brabin BJ. Catch-up growth in Malawian babies, a longitudinal study of normal and low birthweight babies born in a malarious endemic area. *Early Human Development.* 2005; **81**: 841-850.
9. Hauspie RC, Pagezy H. Longitudinal study of growth of African babies: an analysis of seasonal variations in the average growth rate and the effects of infectious diseases on individual and average growth patterns. *Acta Paediatrica Scandinavica. Supplement.* 1989; **350**: 37-43.
10. Johnson W, Darboe MK, Sosseh F, Nshe P, Prentice AM, Moore SE. Association of prenatal lipid-based nutritional supplementation with fetal growth in rural Gambia. *Maternal & Child Nutrition.* 2016; Oct 2. doi: 10.1111/mcn.1236.
11. Goldberg GR, Jarjou LM, Cole TJ, Prentice A. Randomized, placebo-controlled, calcium supplementation trial in pregnant Gambian women accustomed to a low calcium intake: effects on maternal blood pressure and infant growth. *American Journal of Clinical Nutrition.* 2013; **98**: 972-982.
12. van der Merwe LF, Moore SE, Fulford AJ, et al. Long-chain PUFA supplementation in rural African infants: a randomized controlled trial of effects on gut integrity, growth, and cognitive development. *American Journal of Clinical Nutrition.* 2013; **97**: 45-57.
13. Unger S. Effects of physician or health care worker prescribed lipid-based multiple micronutrients on the health status of children presenting to a primary health care centre in The Gambia. PhD Thesis London School of Hygiene and Tropical Medicine; London 2013.
14. Williams EA, Elia M, Lunn PG. A double-blind, placebo-controlled, glutamine-supplementation trial in growth-faltering Gambian infants. *The American journal of clinical nutrition.* 2007; **86**: 421-427.
15. Darboe MK, Thurnham DI, Morgan G, et al. Effectiveness of an early supplementation scheme of high-dose vitamin A versus standard WHO protocol in Gambian mothers and infants: a randomised controlled trial. *Lancet.* 2007; **369**: 2088-2096.
16. Jarjou LM, Prentice A, Sawo Y, et al. Randomized, placebo-controlled, calcium supplementation study in pregnant Gambian women: effects on breast-milk calcium concentrations and infant birth weight, growth, and bone mineral accretion in the first year of life. *American Journal of Clinical Nutrition.* 2006; **83**: 657-666.
17. Krahenbuhl JD, Schutz Y, Jequier E. High fat versus high carbohydrate nutritional supplementation: a one year trial in stunted rural Gambian children. *European Journal of Clinical Nutrition.* 1998; **52**: 213-222.
18. Ceesay SM, Prentice AM, Cole TJ, et al. Effects on birth weight and perinatal mortality of maternal dietary supplements in rural Gambia: 5 year randomised controlled trial. *British Medical Journal.* 1997; **315**: 786-790.
19. Hoare S, Poppitt SD, Prentice AM, Weaver LT. Dietary supplementation and rapid catch-up growth after acute diarrhoea in childhood. *British Journal of Nutrition.* 1996; **76**: 479-490.
20. Bates CJ, Evans PH, Dardenne M, et al. A trial of zinc supplementation in young rural Gambian children. *British Journal of Nutrition.* 1993; **69**: 243-255.
21. Prentice AM, Cole TJ, Foord FA, Lamb WH, Whitehead RG. Increased birthweight after prenatal dietary supplementation of rural African women. *American Journal of clinical Nutrition.* 1987; **46**: 912-925.
